# Full-length transcriptome sequences of *Agropyron cristatum* facilitate the prediction of putative genes for thousand-grain weight in a wheat-*A. cristatum* translocation line

**DOI:** 10.1186/s12864-019-6416-4

**Published:** 2019-12-27

**Authors:** Shenghui Zhou, Jinpeng Zhang, Haiming Han, Jing Zhang, Huihui Ma, Zhi Zhang, Yuqing Lu, Weihua Liu, Xinming Yang, Xiuquan Li, Lihui Li

**Affiliations:** 1 National Key Facility for Crop Gene Resources and Genetic Improvement, Institute of Crop Sciences, Chinese Academy of Agricultural Sciences, Beijing, 100081 China; 2 Rice Research Institute, Guangdong Academy of Agricultural Sciences, Guangzhou, 510640 China

**Keywords:** Full-length transcriptome, Wheat, Wild relative, *Agropyron cristatum*, Gene expression, Thousand-grain weight

## Abstract

**Background:**

*Agropyron cristatum* (L.) Gaertn. (2n = 4x = 28; genomes PPPP) is a wild relative of common wheat (*Triticum aestivum* L.) and provides many desirable genetic resources for wheat improvement. However, there is still a lack of reference genome and transcriptome information for *A. cristatum*, which severely impedes functional and molecular breeding studies.

**Results:**

Single-molecule long-read sequencing technology from Pacific Biosciences (PacBio) was used to sequence full-length cDNA from a mixture of leaves, roots, stems and caryopses and constructed the first full-length transcriptome dataset of *A. cristatum*, which comprised 44,372 transcripts. As expected, the PacBio transcripts were generally longer and more complete than the transcripts assembled via the Illumina sequencing platform in previous studies. By analyzing RNA-Seq data, we identified tissue-enriched transcripts and assessed their GO term enrichment; the results indicated that tissue-enriched transcripts were enriched for particular molecular functions that varied by tissue. We identified 3398 novel and 1352 *A. cristatum*-specific transcripts compared with the wheat gene model set. To better apply this *A. cristatum* transcriptome, the *A. cristatum* transcripts were integrated with the wheat genome as a reference sequence to try to identify candidate *A. cristatum* transcripts associated with thousand-grain weight in a wheat-*A. cristatum* translocation line, Pubing 3035.

**Conclusions:**

Full-length transcriptome sequences were used in our study. The present study not only provides comprehensive transcriptomic insights and information for *A. cristatum* but also proposes a new method for exploring the functional genes of wheat relatives under a wheat genetic background. The sequence data have been deposited in the NCBI under BioProject accession number PRJNA534411.

## Background

As the most widely cultivated crop on Earth, wheat (*Triticum aestivum* L., 2n = 6x = 42, genomes AABBDD) contributes approximately a fifth of the total calories consumed by humans and provides more protein than any other food source [1]. However, due to historical artificial selection and domestication, the genetic diversity of modern wheat is relatively narrow, which is one of the bottlenecks for breakthroughs in wheat improvement [2–4]. Natural variation from collections of wild wheat relatives has been and remains an important facilitator of wheat genetic advances, since these relatives conserve considerable genetic variability of adaptive traits that can be transferred via artificially innovated introgression lines by direct hybridization [5–9].

The genus *Agropyron* Gaertn., called the crested wheatgrass complex, is an out-crossing tertiary gene pool relative of wheat and built upon one basic P genome with 3 ploidy levels [10]. The tetraploid crested wheatgrass *Agropyron cristatum* (L.) Gaertn. (2n = 4x = 28, genome PPPP) not only provides protein as a forage source but also possesses several desirable traits for wheat improvement. In the early 1990s, several wheat-*A. cristatum* derivative lines were produced via the intergeneric hybridization of wheat cv. Fukuhokumugi (Fukuho) and *A. cristatum* accession Z559 and embryo rescue [11]. Several of these lines, including additional lines, disomic substitution lines, translocation lines and introgression lines, exhibit potentially valuable traits for wheat improvement, such as disease resistance, abiotic and biotic stress tolerance and high yield, and these lines have therefore been used in wheat-breeding programmes [12–15]. Among these lines, Pubing 3035, a Ti1AS-6PL-1AS·1AL intercalary translocation, was derived from the offspring of a wheat-*A. cristatum* 6P chromosome addition line; notably, the 6P chromosomal segment played an important role in regulating the thousand-grain weight and spike length [15]. Although the growth characteristics and utilization of wheat-*A. cristatum* derivative lines in wheat-breeding programmes have been extensively investigated, little is known regarding the nature of the gene and the mechanism by which it confers superior traits.

As a result of the low frequency of pairing and suppressed recombination between the genomes of wild wheat relatives and wheat, it is extremely difficult to characterize genes from wheat wild relatives through a map-based cloning strategy under a wheat genetic background. Comprehensive approaches, including cytogenetic stock development, mutagenesis, resistance gene enrichment and sequencing-Pacific Biosciences (PacBio), long-range assembly, and functional analysis, were successively used to successfully clone the *Pm21* gene, which confers high resistance to *Blumeria graminis* f. sp. *tritici* (*Bgt*) in wheat throughout all growth stages, from the wild species *Haynaldia villosa* [16]. At the same time, *Pm21* was also isolated and functionally validated via the discovery of *Bgt*-susceptible *Dasypyrum villosum* resources and construction of a genetic population using resistant intervals [17]. Placido and colleagues identified candidate genes associated with root development from the wheat-*Agropyron elongatum* translocation line by transcriptome analysis, but the relationship between these candidate genes and improved drought adaptation has not yet been elucidated [18]. Most of the studies related to the gene cloning of wild relatives have focused on disease resistance genes, but no relevant studies have reported the cloning of genes associated with complex traits, such as yield-related traits in derived lines. The lack of reference genome sequences severely impedes in-depth molecular breeding and gene functional studies of important wheat wild relatives. Therefore, to reveal the genetic bases of important traits and understand their molecular mechanistic bases, it is particularly urgent to develop an effective strategy for excavating functional candidate genes from wheat and wild relative-derived germplasms expressing superior traits.

RNA-sequencing (RNA-Seq) has recently become a popular technique because it is cost-effective, and it does not rely on a reference genome [19]. RNA-Seq of *A. cristatum* Z559 by the Illumina platform has enabled the successful annotation of orthologous genes related to multiple agronomic traits in *A. cristatum* [20] and has provided many new insights into the phylogenetic relationship and interspecific variation between *A. cristatum* and wheat [21]. However, the short sequencing reads of the Illumina platform make the assembly and annotation of the *A. cristatum* transcriptome incomplete and error-prone. Recently, single-molecule, real-time (SMRT) sequencing technology from PacBio has provided an efficient approach to sequence full-length (FL) cDNA molecules and has been successfully used for whole-transcriptome profiling in many animal and plant species [22–34]. Compared with Illumina and other second-generation sequencing techniques, the advantages of PacBio transcriptome sequencing not only allow complete cDNA sequences containing both the 5′ and 3′ ends to be obtained but also enable identification of alternative isoforms [25, 26].

**Fig. 1:**
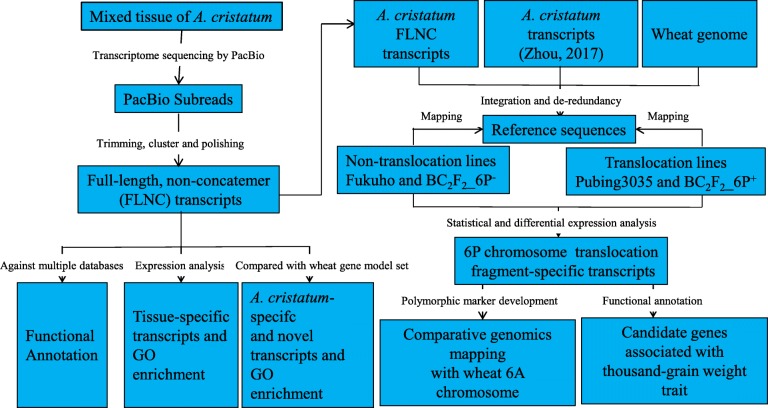
Pipeline for constructing the *A. cristatum* transcriptome and the application of this transcriptome in the identification of candidate alien genes in wheat-*A. cristatum* translocation line Pubing 3035

In this study, we present the first report on the single-molecule FL sequencing, annotation and expression of the *A. cristatum* Z559 transcriptome and the application of this transcriptome in the identification of candidate alien genes associated with thousand-grain weight in the wheat-*A. cristatum* translocation Pubing 3035 (Fig. 1). Single-molecule long-read transcriptome sequencing of *A. cristatum* Z559 was performed using the PacBio Sequel platform, and full-length, non-concatemer (FLNC) transcripts were constructed and annotated. Tissue-specific FLNC transcripts were revealed in *A. cristatum* using RNA-Seq. Then, novel and *A. cristatum*-specific transcripts were identified by comparison with the wheat gene model set. Furthermore, by integrating the *A. cristatum* transcripts, including FLNCs and transcripts assembled in previous studies [21], and the wheat genome as reference sequences, candidate *A. cristatum* transcripts associated with thousand-grain weight were identified in Pubing 3035. The present study not only provides comprehensive transcriptomic insights and information for *A. cristatum* but also proposes a new method for the exploration of functional genes from wheat relatives under a wheat genetic background.

## Methods

### Plant materials

The *A. cristatum* accession Z559 (2n = 4x = 28, PPPP, from Xinjiang, China), a representative tetraploid *A. cristatum*, has been previously described [20] and cultivated in the experimental field of the Chinese Academy of Agricultural Sciences, Beijing, China (E116.33, N39.96). Fukuho, translocation line Pubing 3035 and their BC_2_F_2_ population, which was produced with the recurrent parent Fukuho, were planted in the experimental field of the Chinese Academy of Agricultural Sciences, Xinxiang, Henan province, China (E113.46, N35.8).

### Tissue sampling and RNA isolation

Leaves, stems, roots and caryopses (growth stage 54) from *A. cristatum* plants, leaves and caryopses (growth stage 54, 73, 75 and 77), from Fukuho, Pubing 3035 and their BC_2_F_2_ population, were collected [35]. The samples of *A. cristatum*, Fukuho and Pubing 3035 consisted of tissues from 5 different plants. According to the presence of the translocation fragment, as determined by molecular makers developed by Zhang et al. [14], the BC_2_F_2_ population was divided into two mixed samples each consisting of 30 lines, defined as BC_2_F_2__6P^+^ and BC_2_F_2__6P^−^. All samples were snap-frozen in liquid nitrogen and ground into powder. The total RNA of each sample was extracted using TRIzol Reagent (Invitrogen, Carlsbad, CA, USA) according to the manufacturer’s recommendations. The quantity and integrity of the total RNA were assessed using an Agilent 2100 Bioanalyzer (Agilent Technologies, PaloAlto, CA, USA) and 1% agarose gel electrophoresis. Only total RNA samples with RIN values ≥8 were used for constructing the cDNA libraries.

### Illumina and PacBio RNA-Seq library construction and sequencing

Following the protocol of the Gene Expression Sample Prep Kit (Illumina, San Diego, CA, USA), a total of 15 libraries, namely, 11 libraries from *A. cristatum* leaves, stems and roots (3 biological replicates) and unfertilized caryopses (2 biological replicates) and 4 libraries from Fukuho, Pubing 3035, BC_2_F_2__6P^+^ and BC_2_F_2__6P^−^ mixed RNA from leaves and caryopses from four different periods (no biological replicate), were constructed following the protocol of the Gene Expression Sample Prep Kit (Illumina, San Diego, CA, USA). Then, the 15 libraries were sequenced by Novogene Corporation (Beijing, China) using the Illumina HiSeq 2500 platform with a paired-end read length of 150 bp.

To develop a comprehensive catalogue of transcript isoforms, equal amounts of the total RNA from each sample of *A. cristatum* Z559 were pooled into a single sample and used for PacBio library preparation. Library preparation and sequencing were performed according to the PacBio Iso-Seq protocol by Novogene Corporation (Beijing, China). Two SMRT cells were run on the PacBio sequel platform with non-size-selected RNA from the mixed sample.

Raw PacBio SMRT sequences and Illumina RNA-Seq data for this study have been deposited in the NCBI under BioProject accession number PRJNA534411.

### Subread processing and error correction

Briefly, each sequencing run was processed by *ccs* (https://github.com/PacificBiosciences/ccs) to generate one representative circular consensus sequence (CCS) for each zero-mode waveguide (ZMW). Only ZMWs with at least one full pass (at least one subread with SMRT adapter on both ends) were used for the subsequent analysis. The CCSs were processed to remove primers and unwanted combinations, and sequences were oriented to the 5′-3′ direction using *lima* (https://github.com/pacificbiosciences/barcoding), which offers a specialized *isoseq* mode. Then, to create FLNC transcripts, poly(A) tails were trimmed and artificial concatemers were removed by *refine* in IsoSeq3 (https://github.com/PacificBiosciences/IsoSeq3). The FLNC transcripts were then clustered together using *cluster*. The final polishing step created a consensus sequence for each clustered transcript using *arrow* model in *polish*. BUSCO [36] was used to explore completeness according to conserved orthologue content.

### Functional annotation of FLNC transcripts of *A. cristatum*

Trinotate was used for automatic functional annotation of FLNC transcripts. Trinotate uses a number of different well-referenced methods for functional annotation, including homology search to known sequence data (SwissProt, release 2019_03), protein domain identification (Pfam 32.0) [37], protein signal peptide (signalP version 4, https://www.cbs.dtu.dk/cgi-bin/nph-sw_request?signalp), rRNA (RNAMMER, https://www.cbs.dtu.dk/cgi-bin/sw_request?rnammer) and transmembrane domain (tmHMM version 3.2.1, https://www.cbs.dtu.dk/cgi-bin/nph-sw_request?tmhmm) prediction, and leveraging various annotation databases (eggNOG/GO/Kegg) [38]. The sequence with the best hit was considered the optimal annotation. All functional annotation data derived from the analysis of transcripts was integrated into a SQLite database; SQLite allows terms with specific qualities related to a desired scientific hypothesis to be searched quickly and efficiently and provides a means to create a whole annotation report for a transcriptome (https://github.com/Trinotate/Trinotate.github.io). PLEK (version 1.2), which is a predictor of long non-coding RNAs and messenger RNAs based on k-mer scheme and the support vector machine (SVM) algorithm, was used to distinguish long non-coding RNAs (lncRNAs) from messenger RNAs (mRNAs) [39].

### Analysis of tissue-enriched transcripts

All raw sequence reads from the Illumina sequencing platform were cleaned by removing the RNA adapters and trimming the low-quality bases (Q < 20) with a minimum read length of 36 bases using Trimmomatic (version 0.39) [40]. The cleaned reads of all samples from *A. cristatum* Z559 were mapped to FLNC transcripts using Bowtie2 (version 2.3.5) [41]. The proportion of transcripts with zero coverage and unmapped reads that were not mapped to the transcriptome were counted and used to evaluate the quality of the transcriptome. The fragments per kilobase of transcript per million mapped reads (FPKM) values of the transcripts were calculated using RSEM (version 1.3.1) [42]. “Expressed” transcripts were defined as those with both (1) an average FPKM greater than 4 and (2) a FPKM greater than 2 for each replicate of the given tissue [29]. Significantly differentially expressed transcripts within different tissues were identified using DESeq2 software with a false discovery rate (FDR) < 0.01 and a different expression level log_2_(Fold Change) ≥ 2 (version 3.8) [43]. “Expressed” transcripts that were also significantly differentially expressed in a particular tissue compared to all other tissues were considered tissue-enriched transcripts. The Bioconductor package GOseq (version 3.8) was used to explore functional enrichment among the transcript sets showing tissue-specific expression. Gene Ontology (GO) terms with padj < 0.05 (hypergeometric test) and clusters were plotted using REVIGO [44].

### Comparison of FLNC transcripts of *A. cristatum* and wheat gene model

*A. cristatum* FLNC transcripts were aligned and mapped with GMAP (version 2015-09-29) to the Chinese Spring International Wheat Genome Sequencing Consortium (IWGSC) RefSeq V1.0 reference sequences [45]. Only FLNC transcripts mapping to a single location were retained. Each FLNC transcript mapped to the wheat genome was compared with the existing gene models of IWGSC RefSeq v1.0 annotation by cuffcompare [46]. Transcripts that aligned to intergenic regions of the wheat genome were considered novel transcripts compared with wheat, and transcripts that could not be aligned to the wheat genome were considered *A. cristatum*-specific transcripts. The visualization of the distribution of FLNC transcripts over the IWGSC genome was performed using Circos software (version 0.69–6) [47].

### Discovery of *A. cristatum*-specific genes in the wheat-*A. cristatum* translocation line Pubing 3035

The *A. cristatum* FLNC transcripts, transcripts assembled using short read sequencing [21] and IWGSC wheat RefSeq V1.0 reference sequences [45] were integrated as the reference sequences in this study. To reduce redundancy, the sequences were clustered using CD-HIT-EST with sequence identity set to 100%. Illumina RNA-Seq clean reads from Fukuho, Pubing 3035, BC_2_F_2__6P^+^ and BC_2_F_2__6P^−^ were aligned and mapped to the reference sequences using the STAR tool (version 2.7) [48], using the 2-pass STAR method with a minimum intron length of 20 bp, a maximum intron length of 20 kb and default settings for the other parameters. A raw count matrix containing Pubing 3035, BC_2_F_2__6P^+^, Fukuho and BC_2_F_2__6P^−^ was constructed using the featureCounts program [49]. Significant differences in the read counts of transcripts between translocation lines (Pubing 3035 and BC_2_F_2__6P^+^) and non-translocation lines (Fukuho and BC_2_F_2__6P^−^) were detected by the package DESeq2 [43]. The output of DESeq2 consisted of the transcript IDs, base mean values, log_2_(fold change) for translocation versus non-translocation, standard error (IfcSE) values, Wald statistic values, Wald test *P* values and adjusted P values. The transcripts from *A. cristatum*, including FLNC and Trinity-assembled transcripts, that were found to have a log_2_(fold change) ≤ − 4 and adjusted P value ≤0.05 were considered to be from the translocation fragment of Pubing 3035. The transcripts from the translocation fragment of Pubing 3035 were used to search the IWGSC Chinese Spring annotation to find homologous genes for polymorphic marker development. BatchPrimer3 was used to design primer pairs [50]. PCR amplification was carried out on the DNA of *A. cristatum* Z559, Pubing 3035 and Fukuho. PCR products were separated in 8% non-denaturing polyacrylamide gels, visualized by silver staining and photographed.

## Results

### Construction and annotation of the FLNC transcriptome database for *A. cristatum*

**Fig. 2:**
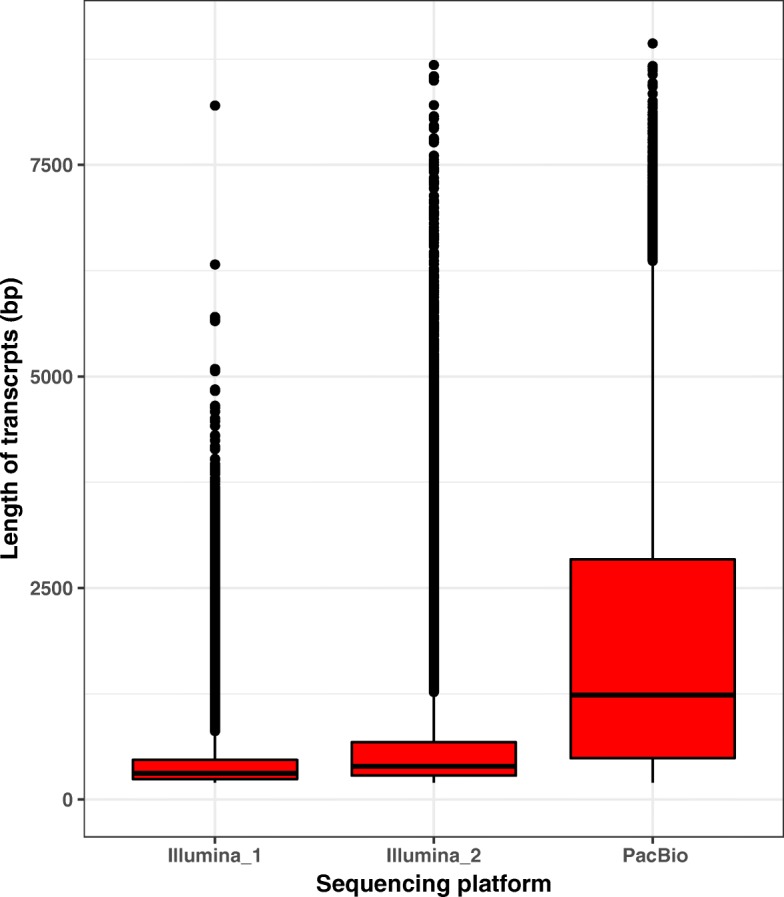
Length distribution of transcripts obtained by different sequencing platforms. Illumina_1 and Illumina_2 represent the transcripts assembled by Zhang [20] and Zhou [21], respectively, using the Illumina sequencing platform

After quality control, a total of 11,966,252 subreads, namely, 6,447,695 and 5,518,557 subreads from two different cells, were successfully generated (Table 1). A total of 504,811 representative CCSs for ZMWs were obtained. A total of 405,302 CCSs were classified as FL transcripts based on the presence of 5′ primers, 3′ primers and poly(A) tails. After demultiplexing, refining, clustering and polishing of FL transcripts were performed, a total of 44,372 FLNC transcripts with a maximum length of 9468 bp, a N50 of 3572 bp and average FL coverage of 5.1 were generated (Table 1). In addition, the proportion of incomplete transcripts of FLNC transcripts was only 6.30% in BUSCO analysis (Table 2). As expected, the PacBio FLNC transcripts were generally longer and more complete than the transcripts assembled via the Illumina sequencing platform in previous studies [20, 21] (Fig. 2; Table 2). However, the higher proportion of unmapped reads (72.24%) indicated that PacBio could not detect all transcripts due to insufficient sequencing data (Table 2). These results indicated that the PacBio FLNCs and transcripts assembled by 2nd generation sequencing should be integrated to obtain a high-quality *A. cristatum* transcriptome database.

**Table 1:** Statistics of different kinds of *A. cristatum* SMRT sequencing reads

Category	First cell	Second cell
No. of subreads	6,447,695	5,518,557
No. of CCS	260,305	244,506
No. of FL transcripts	208,321	196,981
No. of FLNC transcripts	201,518	190,834
No. of FLNC transcripts after merged	392,352	
No. of FLNC transcripts after clustered and polished	44,372	
Average full-length coverage	5.1	
Maximum FLNC reads length (bp)	9468	
Average transcript length (bp)	1874	
N50 length (bp)	3572	

Notes: *CCS* represents circular consensus sequence; *FLNC* represents full-length, non-concatemer

**Table 2:** Statistical comparison of transcriptome assembled by different sequencing platforms

	Proportion of non-existing transcripts^c^	Proportion of unmapped reads^d^	BUSCO analysis with fragment ratio^e^	N50 (bp)
Illumina_1^a^	7.35%	18.28%	31.10%	1026
Illumina_2^b^	23.3%	48.02%	8.80%	651
PacBio	13.67%	72.24%	6.30%	3572

Notes: ^a^ represents transcripts assembled by Zhang [20] using the Illumina GAII sequencing platform; b represents transcripts assembled by Zhou [20] using the Illumina HiSeq 2500 sequencing platform; ^c^ represents the proportion of transcripts with zero coverage after realignment of reads on the transcriptome; ^d^ represents the proportion of unmapped reads that were not mapped to the transcriptome; ^e^ represents the proportion of incomplete transcripts in the BUSCO analysis

**Fig. 3:**
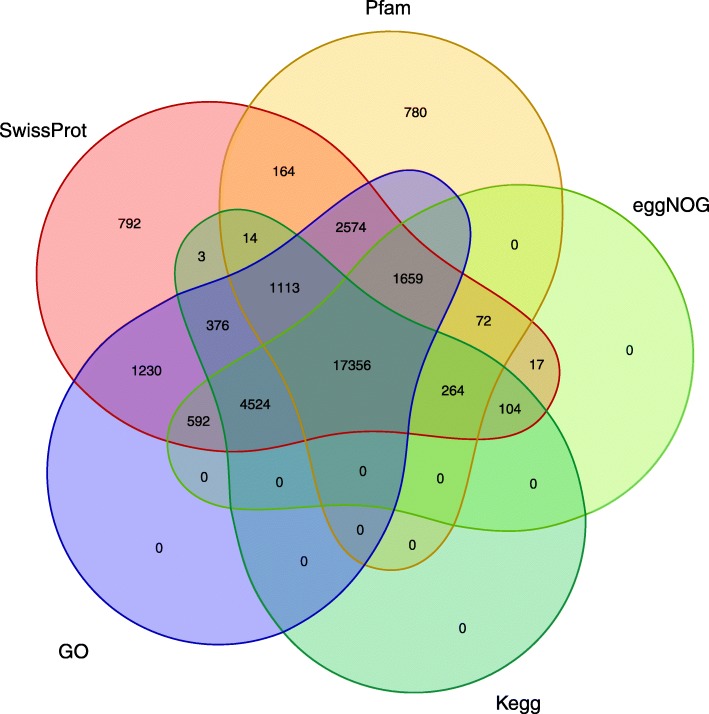
Venn diagram showing the overlap of Pfam, SwissProt, eggNOG, GO and Kegg annotations of *A. cristatum* FLNC transcripts

Functional annotation of the FLNC transcripts was conducted using 5 different public databases (Table 3; Fig. 3). Of these, 30,854 FLNC transcripts were found to have homologs in the SwissProt database. A total of 24,588 transcripts had significant matches in the eggNOG database, and 23,996 transcripts received Pfam domain assignments. Furthermore, 23,754 transcripts had matches in the Kegg database, and 29,424 transcripts were associated with GO terms. Moreover, the numbers of FLNC transcripts with transmembrane regions, signal peptides and rRNA transcripts were 5601, 2344 and 329, respectively. Altogether, 32,318 FLNC transcripts had at least one annotation (Table 3). In addition to protein-coding RNAs, 8202 candidate non-coding RNAs were predicted in non-annotated FLNC transcripts.

**Table 3:** Statistics on functional annotations of the *A. cristatum* FLNC transcripts

Category	No.	Ratio
FLNC transcripts	44,372	100.0%
FLNC transcripts with blast hits to SwissProt	30,854	69.5%
FLNC transcripts with blast hits to eggNOG	24,588	55.4%
FLNC transcripts with blast hits to Pfam	23,996	54.1%
FLNC transcripts with blast hits to Kegg	23,754	53.5%
FLNC transcripts with GO terms	29,424	66.3%
FLNC transcripts with transmembrane regions	5601	12.6%
FLNC transcripts with signal peptides	2344	5.3%
FLNC transcripts with rRNA transcripts	329	0.7%
FLNC transcripts with at least one annotation	32,318	72.8%
FLNC transcripts with non-coding sequences	8202	18.5%

### Tissue-enriched FLNC isoforms

**Fig. 4:**
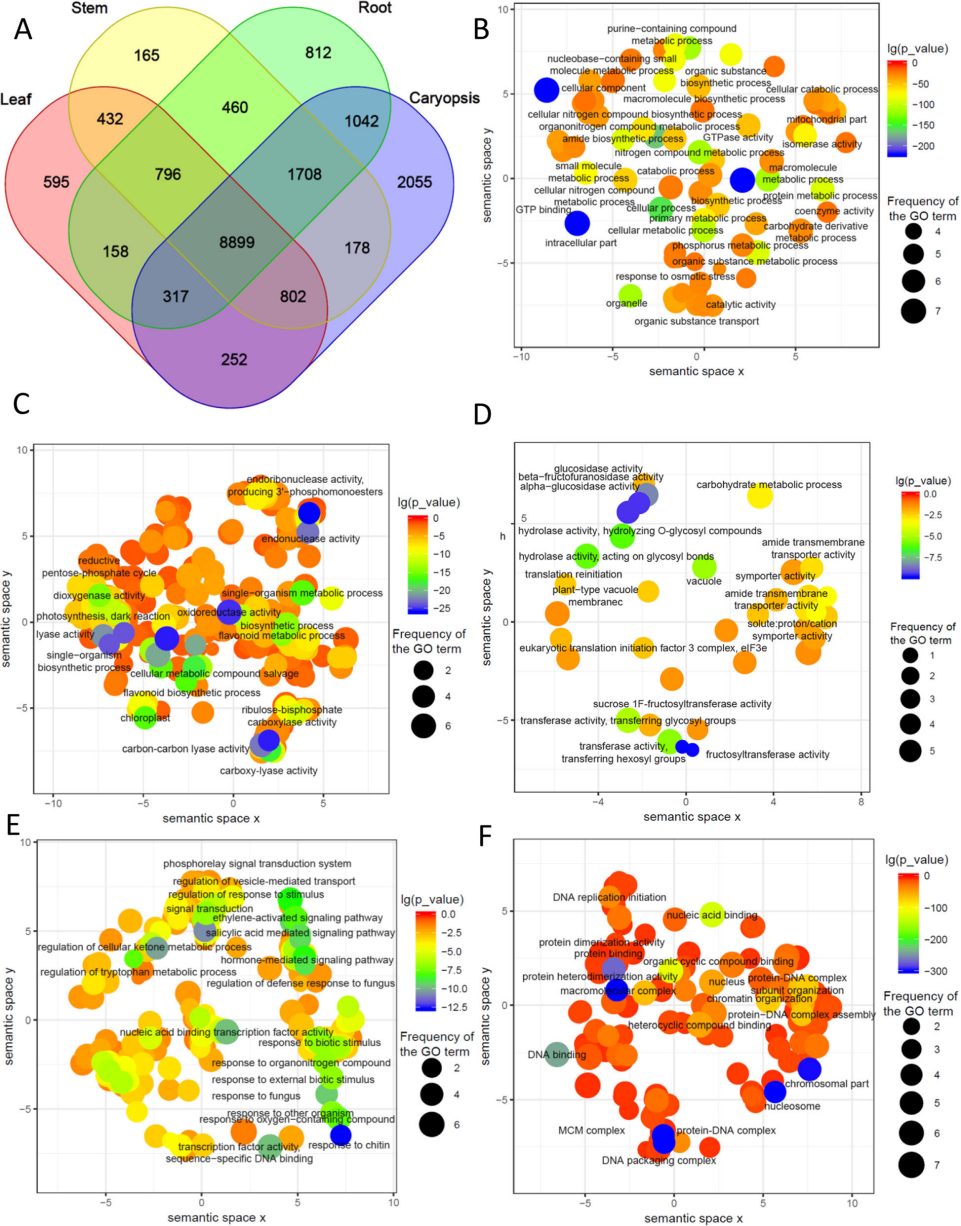
Tissue expression analysis of *A. cristatum* FLNC transcripts. **a**, Number of the protein-coding transcripts expressed in each sampled tissue. **b**, GO enrichment analysis for transcripts expressed in all sampled tissues. **c**, GO enrichment analysis for transcripts enriched in leaves. **d**, GO enrichment analysis for transcripts enriched in stems. **e**, GO enrichment analysis for transcripts enriched in roots. **f**, GO enrichment analysis for transcripts enriched in caryopses

To analyse tissue-enriched transcript expression, a total of 11 transcriptome libraries were generated from 4 different tissues with multiple biological replicates of *A. cristatum* (Additional file 1: Table S1). The Illumina sequencing generated approximately 15 million sequencing reads in each sample. After filtering the low-quality reads, about 99.98% of the sequencing reads were retained for downstream analysis. Quality-controlled RNA-Seq reads from the leaves, stems, roots and caryopses of *A. cristatum* were mapped to FLNC transcripts (Additional file 1: Table S1). “Expressed” transcripts were defined as those with both (1) an average FPKM greater than 4 and (2) an FPKM greater than 2 for each replicate of the given tissue [29], resulting in the detection of 12,251 leaf, 13,440 stem, 14,192 root and 15,253 caryopsis protein-coding transcripts and 8899 transcripts that may have “housekeeping” functions and were expressed in all sampled tissues (Fig. 4a). As expected, GO enrichment analysis showed that basic cell biological and metabolic processes were enriched in the 8899 ubiquitously expressed transcript set, including terms such as organonitrogen compound metabolic and biosynthetic process, organic substance metabolism, protein and peptide metabolism, and amide metabolic and biosynthetic based process (Fig. 4b; Additional file 2: Table S2). Additionally, the ubiquitous category shared intracellular part, organelle, ribonucleoprotein complex, and mitochondrial part terms.

Tissue-enriched transcripts, that is, transcripts expressed at significantly higher levels in a particular tissue compared to all other tissues (FDR ≤0.01, Fold Change ≥4, FPKM ≥2) were next identified in each type of tissue. We observed that the caryopsis tissue had the highest number of tissue-enriched transcripts (1515), followed by leaf (266), root (210), and stem (32) tissues. As expected, GO analysis showed that tissue-enriched FLNC transcripts were enriched for particular molecular functions that varies with tissues. Leaf tissue-enriched transcripts were associated with photosynthesis, with GO terms such as oxidoreductase activity, ribulose-bisphosphate carboxylase activity, photosynthesis dark reaction, carbon-carbon lyase activity, chloroplast, and flavonoid biosynthetic process. (Fig. 4c; Additional file 3: Table S3). In addition, the stem tissue-enriched set was associated with many well-characterized transporter activity functions, including transferase activity, transferring glycosyl groups, transferring hexosyl groups, sucrose 1F-fructosyltransferase activity, fructosyltransferase activity, peptide:proton symporter activity, solute:proton symporter activity, solute:cation symporter activity, amide transmembrane transporter activity, symporter activity, and proton-dependent peptide secondary active transmembrane transporter activity GO terms (Fig. 4d; Additional file 4: Table S4). GO enriched analysis of the root tissue suggested that, in addition to expected categories associated with response to stress (response to external biotic stimulus, response to fungus, and response to biotic stimulus, regulation of defence response to fungus, and regulation of response to stimulus) and signal transduction (hormone-mediated signalling pathway, salicylic acid mediated signalling pathway, ethylene-activated signalling pathway and phosphorelay signal transduction system), response to chitin, oxygen-containing compound, and organonitrogen compound terms appeared in the root-enriched transcript list (Fig. 4e; Additional file 5: Table S5). The vast majority of GO terms associated with the caryopsis tissue-enriched genes were related to cellular processes, including protein heterodimerization activity, protein-DNA complex, DNA packaging complex, nucleosome, chromosomal part, protein dimerization activity, and DNA/nucleic acid/protein binding/heterocyclic compound binding terms (Fig. 4f; Additional file 6: Table S6). In summary, these tissue-enriched GO terms may provide insight into gene expression in *A. cristatum* tissue development and maintenance.

### FLNC transcripts compared with wheat gene model

**Fig. 5:**
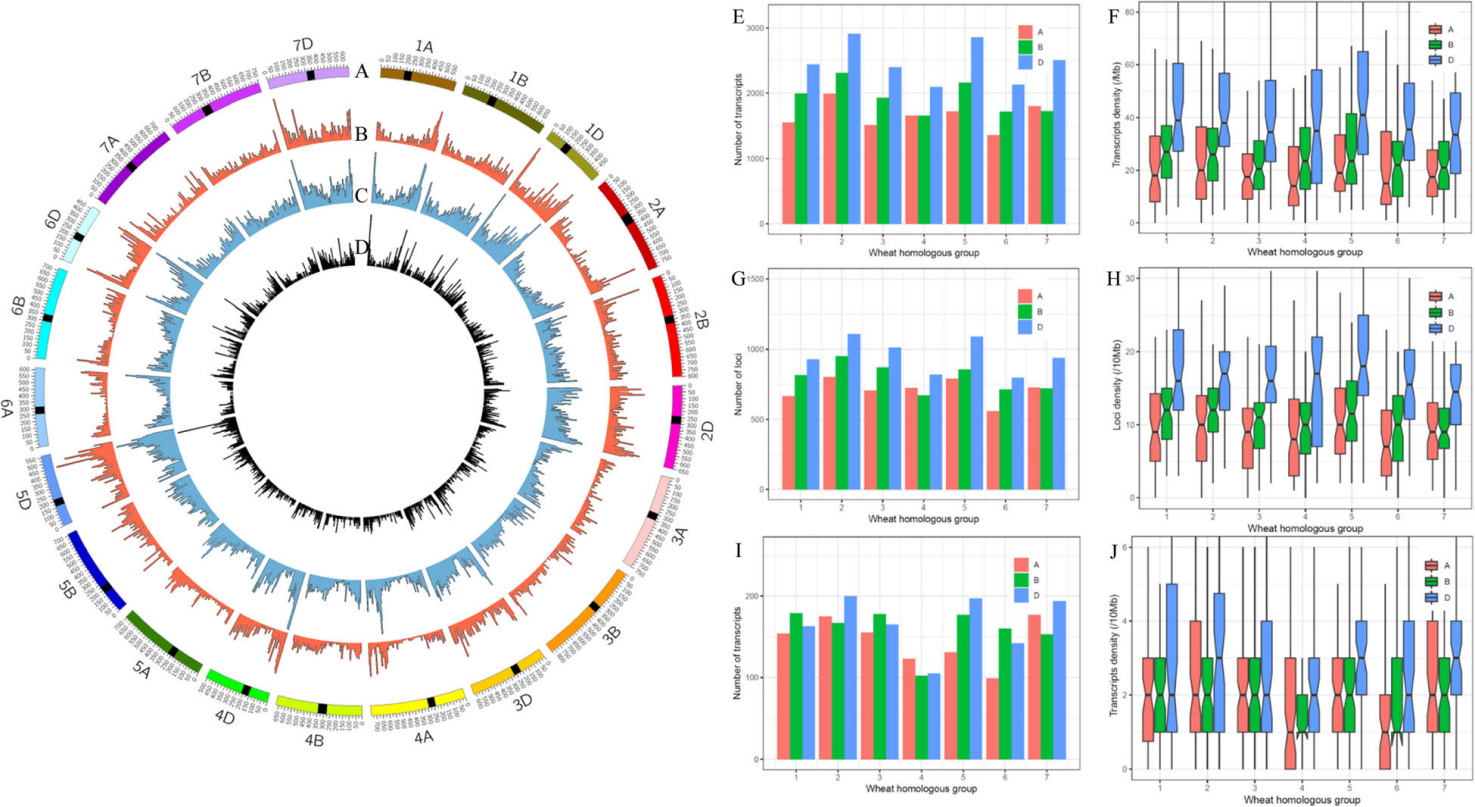
Distribution of orthologues of *A. cristatum* transcripts in the wheat genome. **a**, Karyotype of the wheat genome. The putative pericentromeric-centromeric regions are marked with black [51]. **b**, *A. cristatum* FLNC transcript density distribution; transcript density was calculated in a 10-Mb window. **c**, Loci density distribution; loci density was calculated in a 10-Mb window. **d**, Novel transcript density distribution; transcript density was calculated in a 10-Mb window. **e**, Distribution of FLNC transcripts in the wheat genome. **f**, Box plot of transcript density in the wheat genome. **g**, Distribution of loci in the wheat genome. **h**, Box plot of loci density in the wheat genome. **i**, Distribution of novel transcripts in the wheat genome. **j**, Box plot of novel transcript density in the wheat genome

To compare transcripts between *A. cristatum* and wheat, the 44,372 FLNC transcripts were aligned to the IWGSC RefSeq v1.0 genome (Fig. 5) and compared with the wheat gene set model. A total of 43,020 FLNC transcripts were mapped to 17,510 loci that were spread across the wheat genome (Fig. 5b and c). Among these transcripts, 16,374 FLNC transcripts had multiple exons, and 4604 loci had multiple transcripts, with an average of 1.8 transcripts per locus. The distribution and density of FLNC transcripts on the wheat genome were calculated for all chromosomes in the wheat genome, and sharply decreased from the telomeres to centromeres in the whole wheat chromosomal regions (Fig. 5b). The number of FLNCs in each chromosome was not directly proportional to the chromosomal length and gene number. The most FLNC transcripts were aligned to the homologous group 2 chromosomes, whereas the homologous group 6 chromosomes contained the fewest FLNC transcripts (Fig. 5e). Interestingly, the highest FLNC transcript number and density were observed on the wheat D genome (17,326, 43.5 FLNC transcripts/10 Mb), followed by the wheat B genome (13,500, 25.8 FLNC transcripts/10 Mb), and the wheat A genome (11,612, 23.4 FLNC transcripts/10 Mb) (Fig. 5b, e and f). The distribution and density of the wheat-genome loci to which FLNC transcripts were mapped were similar to the distribution and density of FLNC transcripts (Fig. 5c, g and h). In total, 3398 novel FLNC transcripts were mapped to the intergenic regions of the wheat genome that did not overlap with wheat genes (Fig. 5d and i). The density of the novel transcripts also decreased from the chromosome ends towards the centromeres (Fig. 5d), and the highest density was also observed in the wheat D genome (Fig. 5j).

**Fig. 6:**
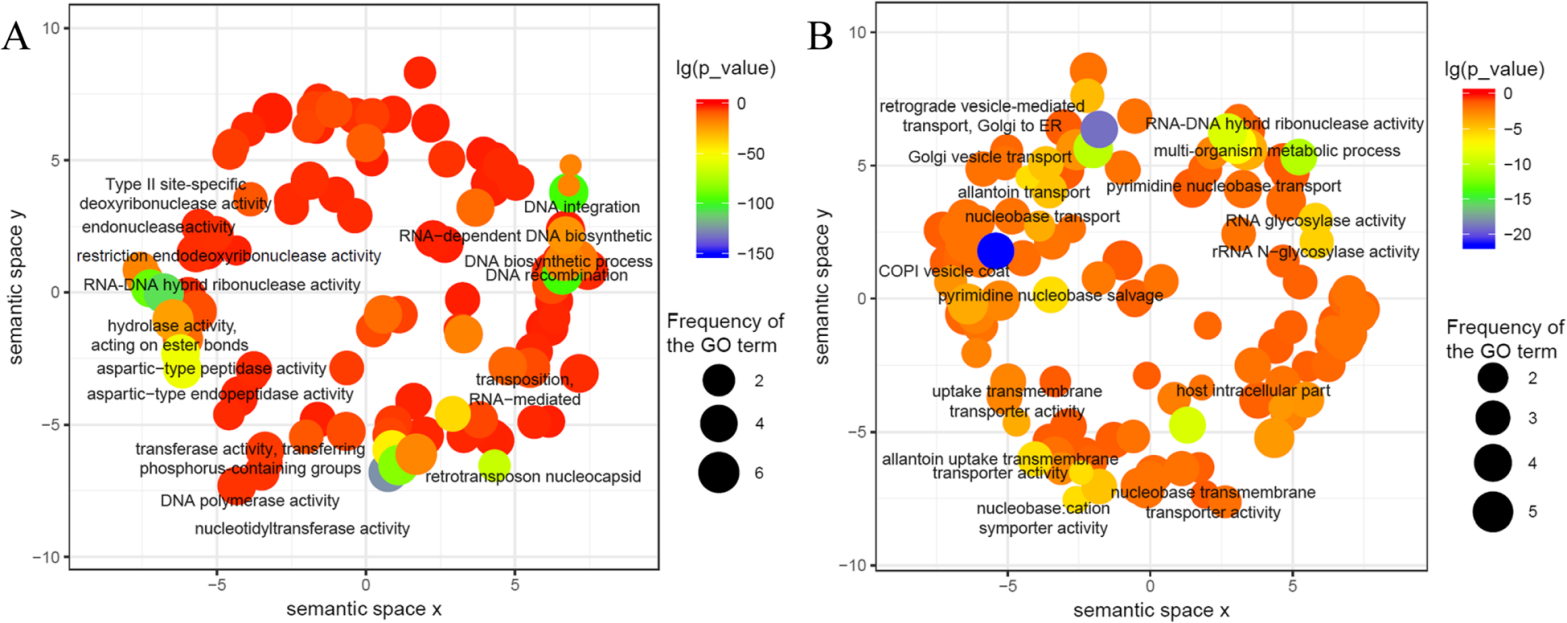
GO enrichment analysis for novel and *A. cristatum*-specific transcripts. **a**, GO enrichment analysis for novel transcripts. **b**, GO enrichment analysis for *A. cristatum*-specific transcripts

GO analysis showed that novel FLNC transcripts were enriched for nucleic acid biological activity and biosynthetic processes, such as DNA polymerase activity, endonuclease activity, DNA recombination, integration and DNA biosynthetic processes, RNA-DNA hybrid ribonuclease activity, and nucleotidyltransferase activity (Fig. 6a; Additional file 7: Table S7). In addition, there were 1352 FLNC transcripts that were not aligned to the wheat genome that are considered to be *A. cristatum*-specific transcripts compared with wheat. The vast majority of GO terms associated with the *A. cristatum*-specific transcripts, including the COPI vesicle coat, retrograde vesicle-mediated transport from Golgi to endoplasmic reticulum, and Golgi vesicle transport terms, were related to protein transport processes in the cytoplasm. Additionally, these transcript categories shared terms associated with multi-organism metabolic processes such as the RNA-DNA hybrid ribonuclease activity, transporter activity of nucleobase:cation symporter, uptake transmembrane and nucleobase transmembrane terms (Fig. 6b; Additional file 8: Table S8). Thus, these 4750 FLNC transcripts, containing 3398 novel and 1352 *A. cristatum*-specific transcripts, might represent particularly positive selection compared with wheat and be helpful for understanding the genetic diversity of *Triticeae*.

### Identification of candidate genes associated with thousand-grain weight in *A. cristatum*-wheat translocation line Pubing 3035

A total of 4 transcriptome libraries were generated from mixed RNA from the leaves and caryopses of Fukuho, Pubing 3035, BC_2_F_2__6P^+^ and BC_2_F_2__6P^−^ sampled during four different periods. Illumina sequencing generated 42,700,222, 30,258,610, 29,705,108 and 30,538,203 sequence reads in Fukuho, Pubing 3035, BC_2_F_2__6P^+^ and BC_2_F_2__6P^−^, respectively. After filtering the low-quality reads, approximately 99.98% of the sequencing reads (42,692,045 reads for Fukuho, 30,253,410 reads for Pubing 3035, 29,699,108 reads for BC_2_F_2__6P^+^ and 30,533,230 for BC_2_F_2__6P^−^) were retained for downstream analysis (Additional file 1: Table S1).

**Fig. 7:**
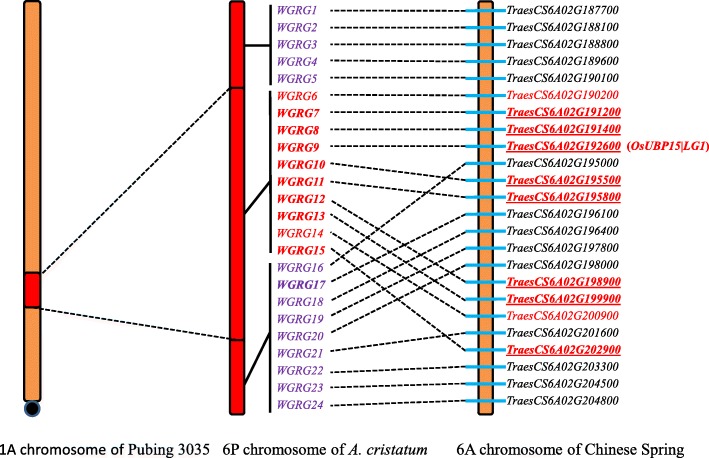
Comparative genomics map between the 6P chromosome translocation fragment in Pubing 3035 and the 6A chromosome of wheat

To identify genes specifically expressed in the translocation fragment, high-quality clean sequencing data were aligned to the reference sequences from an integration of the *A. cristatum* transcriptome and the wheat genome. Differential analysis using DESeq2 revealed that a total of 12 *A. cristatum* transcripts exhibited differential expression between non-translocation and translocation lines that met the parameters of log_2_(fold change) ≤ − 4 and adjusted *P* value ≤0.05 (Table 4). The sequences of these 12 significantly differentially expressed transcripts were used as queries to search orthologous regions from genome sequences of wheat; the search indicated that homologous genes were located in the same interval on chromosome 6A/B/D. These intervals ranged from the *TraesCS6A02G191200* gene to the *TraesCS6A02G202900* gene on chromosome 6A, spanning 82.8 Mbp, from the *TraesCS6B02G219700* gene to the *TraesCS6B02G233700* gene on chromosome 6B, spanning 80.9 Mbp, and from the *TraesCS6D02G174400* gene to the *TraesCS6D02G187400* gene on chromosome 6D, spanning 88.7 Mbp (Additional file 9: Table S9). transcript/24685 and TRINITY_DN94508_c0_g1_i1, transcript/16718 and TRINITY_DN118140_c0_g2_i2, transcript/14210 and transcript/9968 and TRINITY_DN12662_c0_g1_i1 and TRINITY_DN75295_c0_g1_i1 corresponded to the same homologs of the wheat genome, suggesting that they might be isoforms of the same gene or be derived from different homologous genes (Additional file 9: Table S9). We developed polymorphic markers based on the sequences of homologous genes in the wheat 6A/B/D chromosomal regions corresponding to the 12 differentially expressed transcripts (Tables 4; Additional file 10: Table S10; Additional file 11: Figure S1). The orthologous genomic regions of the translocation fragment in *A. cristatum* were identified in wheat chromosome 6A (Fig. 7), indicating that the wheat chromosome interval corresponding to the *A. cristatum* translocation fragment in Pubing 3035 was from the *TraesCS6A02G190200* to the *TraesCS6A02G204000* gene of chromosome 6A and that obvious rearrangements could be observed on the 6P translocation segment compared with the wheat 6A chromosome (Fig. 7). According to these results, it could be speculated that the genomic region of the translocation fragment in *A. cristatum* shows collinearity with chromosomes 6A of wheat.

**Table 4:** Statistics of the analysis of 6P translocation fragment-specific transcripts in Pubing 3035

Transcript ID	Fukuho (6P^−^) read count	BC_2_F_2__6P^−^ read count	BC_2_F_2__6P^+^ read count	Pubing 3035 (6P+) read count	log_2_FC^a^	lfcSE^b^	stat^c^	Pvalue^d^	padj^e^	Polymorphic markers
transcript/2610	0	2	86	127	−7.04	1.18	−5.95	2.62E-09	2.30E-05	*WGRG7*
transcript/24685	1	2	691	372	−8.79	0.96	−9.12	7.52E-20	1.55E-15	*WGRG8*
TRINITY_DN94508_c0_g1_i1	0	1	74	48	−7.25	1.58	−4.59	4.42E-06	9.73E-03	*WGRG8*
transcript/7882	0	0	27	26	−7.49	1.77	−4.23	2.31E-05	3.84E-02	*WGRG9*
transcript/29056	0	0	28	47	−7.99	1.71	−4.67	3.03E-06	8.11E-03	*WGRG10*
transcript/34773	0	0	144	174	−10.08	1.53	−6.59	4.29E-11	5.29E-07	*WGRG11*
transcript/16718	1	1	34	56	−5.82	1.28	−4.53	5.87E-06	1.17E-02	*WGRG12*
TRINITY_DN118140_c0_g2_i2	0	0	64	118	−9.27	1.60	−5.80	6.65E-09	3.73E-05	*WGRG12*
transcript/14210	0	0	69	119	−9.32	1.59	−5.86	4.50E-09	3.47E-05	*WGRG13*
transcript/9968	0	0	30	39	−7.87	1.71	−4.60	4.16E-06	9.49E-03	*WGRG13*
TRINITY_DN12662_c0_g1_i1	0	0	64	87	−9.00	1.59	−5.66	1.49E-08	7.09E-05	*WGRG15*
TRINITY_DN75295_c0_g1_i1	0	1	52	60	−7.13	1.57	−4.53	5.85E-06	1.17E-02	*WGRG15*

Notes: ^a^ represents log_2_(fold change) for translocation lines (Pubing 3035 and BC_2_F_2__6P^+^) versus non-translocation lines (Fukuho and BC_2_F_2__6P^−^); ^b^ represents standard error; ^c^ represents Wald statistic; ^d^ represents Wald test P value; ^e^ represents adjusted *P* values

The functions of these 12 significantly differentially expressed transcripts were investigated and one of them, transcript/7882, was homologous to the rice gene *OsUBP15*/*LG1*, which encodes a constitutively expressed ubiquitin-specific protease 15 (*OsUBP15*) that possesses de-ubiquitination activity in vitro and is a positive regulator of grain width and size in rice [52]. The marker *WGRG9* was developed from transcript/7882 and corresponding with *TraesCS6A02G192600*, *TraesCS6B02G231700* and *TraesCS6D02G179700* (Fig. 7; Additional file 9: Table S9). Therefore, the orthologue of *WGRG9* in the corresponding P genomic region can serve as a candidate gene for control of thousand-grain weight in Pubing 3035; this gene should be subjected to functional verification in a future study.

## Discussion

To broaden the wheat gene pool and provide new potential genes for wheat breeding, many desirable genes from *A. cristatum* have been transferred into common wheat by the intergeneric hybridization of common wheat and *A. cristatum* [53]. However, the progress in *A. cristatum* genomic studies lags far behind the production and application of wheat–*A. cristatum* derivatives, limiting our understanding of the genetic effects of alien fragments/genes on agronomic traits and the application of these derivative lines to wheat breeding projects [54]; one of the main reasons for this lack of understanding is that the reference genome of *A. cristatum* is not currently available because of its large size and high heterozygosity. Therefore, the construction of a FL cDNA sequence database for *A. cristatum* is critically important to fully unveil the molecular mechanisms of alien genes in wheat-*A. cristatum* novel germplasms until the whole-genome sequencing of *A. cristatum* has been achieved. In this study, a FL transcriptome database of *A. cristatum* was constructed using PacBio single-molecule technology, which provided useful information and showed three major features, which are described as follows.

### Integration of PacBio FLNCs and transcripts assembled by 2nd generation sequencing in complex de novo transcriptome analysis

High-throughput transcriptome sequencing has recently become a popular technique because it is cost-effective, does not rely on a reference genome and can contribute to transcriptional analysis, molecular marker development and gene discovery [19]. In a previous study, de novo transcriptome assembly and unigene functional annotation were conducted in *A. cristatum* with the Illumina sequencing technique, and gene resources that were related to traits of interest and specific to *A. cristatum* within the tribe Triticeae, as well as the phylogenetic relationship and interspecific variation between *A. cristatum* and wheat, were identified [20, 21]. However, transcriptome analyses in species without reference genome sequences often encounter complicated problems, especially in the assembly of sequencing reads; thus, the assembly and annotation of *A. cristatum* is incomplete and error-prone (Table 2), which severely impedes in-depth molecular breeding and gene functional studies of *A. cristatum*. Compared to Illumina RNA-Seq, PacBio SMRT-Seq has the ability to assemble FL transcripts due to its longer read length and zero need for PCR amplification during library construction. Therefore, we analysed mixed samples of leaves, stems, roots and caryopses with single-molecule long-read sequencing technology from PacBio and reported the first FL transcriptome dataset of *A. cristatum* comprising 44,372 FLNC transcripts. Compared with the assembled transcripts of *A. cristatum* obtained from the Illumina sequence platform in previous studies, the average length and integrity of the transcripts in this study were greatly increased (Fig. 2; Table 2). However, the higher proportion of unmapped reads indicated that PacBio could not detect all transcripts due to insufficient sequencing data (Table 2). Therefore, PacBio FLNCs and transcripts assembled by 2nd generation sequencing should be integrated to obtain a high-quality *A. cristatum* transcriptome database in complex de novo transcriptome analysis. The integrated transcriptome database will provide resources for the study of gene expression and the discovery and development of specific markers in *A. cristatum*.

### Tissue-enriched transcript expression and enrichment analysis of *A. cristatum*

The study of tissue-specific genes will provide insights into tissue development and evolution and has been verified in several plant species. MacMillan used cotton as a model to study different secondary cell walls and the expression of the genes involved in their formation via RNA deep sequencing of the stem and seed fibre, revealing the subtleties of the gene regulation underlying the diversity of plant secondary cell walls [55]. To comprehensively annotate genes in the yerba mate phenylpropanoid pathway and to evaluate differential expression profiles, Fay generated tissue specific transcriptomic profiles [56]. The combination of reference sequences for FLNC transcriptomes and RNA-Seq technology allowed the identification of tissue-specific expressed transcripts of *A. cristatum*. A comprehensive transcriptome profile of the major tissue types comprising the ovary and young fruit of tomato was obtained using laser-capture microdissection and RNA-Seq, revealing great diversity in gene expression associated with tissue type and developmental stage [57]. In this study, we performed RNA-Seq profiling of gene expression in four tissues of *A. cristatum*. By mapping the FLNC transcripts in this study, we identified tissue-specific expressed transcripts and enriched GO terms using statistical and gene set enrichment methods. A total of 266, 210, 32 and 1515 tissue-enriched transcripts were identified in leaves, roots, stems and caryopses, respectively. As expected, GO analysis showed that tissue-enriched FLNC transcripts were enriched for particular molecular functions that varies with tissues. Leaf tissue-enriched genes were associated with photosynthesis (Fig. 4c; Additional file 3: Table S3). The stem tissue-enriched set was associated with many well-characterized transporter activity functions (Fig. 4d; Additional file 4: Table S4). GO enrichment analysis suggested that in addition to expected categories associated with responses to stress and signal transduction, terms associated with responses to chitin, oxygen-containing compounds, and organonitrogen compounds appeared in the root-enriched transcripts list (Fig. 4e; Additional file 5: Table S5). The vast majority of GO terms associated with the caryopsis tissue-enriched genes were related to cellular processes (Fig. 4f; Additional file 6: Table S6). The isolation and RNA-Seq analysis of four major tissues of *A. cristatum* revealed that the transcriptomes of tissues differ from one another, highlighting the importance of transcriptionally profiling specific developmental tissues to understand the corresponding biology. These data provide resources to explore the application of *A. cristatum* genes in wheat breeding.

### An effective strategy for excavating functional candidate genes from wheat and wild relative-derived germplasms expressing a given trait

For many decades, desirable traits have been transferred from wild relatives into wheat through a series of wide crossings by cytogeneticists and breeders. However, because of the suppressed recombination between the alien chromatin and the domesticated chromatin, alien introgressions, which are an important source of genetic variation in wheat breeding, have fallen into disfavour with many breeders due to the co-introduction of undesirable alleles of genes on the alien introgression, a process known as linkage drag [58]. Therefore, the desirable genes related to the target traits should be separated from the linked sequences when introducing alleles between wheat and wild relatives. Advanced biotechnologies, such as next-generation sequencing and homology-based cloning, have proven beneficial in accelerating gene discovery directly from derived lines of wheat and wild relatives [16, 59, 60]. However, most of these studies related to the cloning of genes from derived lines have focused on disease-resistant genes, and no relevant studies have reported the cloning of genes associated with complex traits such as yield directly from derived lines. Thus, new strategies need to be developed to fully access and exploit the rich gene source found in the wild relatives of wheat under a wheat background. In this study, by integrating transcriptome databases of a wild wheat relative, *A. cristatum*, and wheat genome sequences into a comprehensive reference sequence, we effectively excavated functional candidate genes from their translocation line. Furthermore, we reported the successful application of this strategy in the excavation of wild relative-specific genes from the wheat and *A. cristatum*-derived translocation line Pubing 3035, in which the alien chromosome fragment has a positive regulatory effect on thousand-grain weight and spike length in wheat [15]. A total of 12 *A. cristatum* transcripts were identified as differentially expressed and verified by PCR experiments between the non-translocation and translocation lines, and their homologous genes were located in the same interval on chromosome 6A/B/D. Previous studies have shown that the P genome is more closely related to wheat genome A than to the B and D genomes. Therefore, a comparative genomics map between the 6P chromosome fragment in Pubing 3035 and the wheat 6A chromosome was constructed by developing polymorphic molecular markers of conserved homologous genes between the 6P and 6A chromosomes. The results show that *A. cristatum* transcript/7882 corresponds with *TraesCS6A02G192600*, *TraesCS6B02G231700* and *TraesCS6D02G179700* (Fig. 7; Additional file 9: Table S9) and is homologous to rice gene *OsUBP15*/*LG1*, which encodes a constitutively expressed ubiquitin-specific protease 15 (OsUBP15) that possesses de-ubiquitination activity in vitro and is a positive regulator of grain width and size in rice [52], suggesting that their orthologue in the corresponding P genomic region could serve as a candidate gene for controlling thousand-grain weight in Pubing 3035. The effective research method used in this study can be applied in other studies to discover candidate genes in wheat and wild relative-derived translocation lines with prominent traits.

## Conclusion

Single-molecule long-read transcriptome sequencing of *A. cristatum* Z559 was performed using the PacBio Sequel platform. A total of 44,372 FLNC transcripts were constructed and annotated. Tissue-enriched FLNC transcripts were revealed in *A. cristatum* using RNA-Seq. Then, novel and *A. cristatum*-specific transcripts were identified by comparison with the wheat gene model set. Furthermore, by integrating the *A. cristatum* transcripts with the wheat genome as a reference sequence, 12 candidate *A. cristatum* transcripts associated with thousand-grain weight were identified in Pubing 3035 and verified to be genuine via polymorphic molecular markers. The present study not only provides comprehensive transcriptomic insights and information for *A. cristatum* but also proposes a new method for the exploration of functional genes from wheat relatives under a wheat genetic background.

## Supplementary information

**Additional file 1: Table S1.** Summary of the Illumina sequencing data.

**Additional file 2: Table S2.** GO enrichment analysis for transcripts expressed in all sampled tissues.

**Additional file 3: Table S3**. GO enrichment analysis for transcripts enriched in leaves.

**Additional file 4: Table S4**. GO enrichment analysis for transcripts enriched in stems.

**Additional file 5: Table S5**. GO enrichment analysis for transcripts enriched in roots.

**Additional file 6: Table S6**. GO enrichment analysis for transcripts enriched in caryopses.

**Additional file 7: Table S7**. GO enrichment analysis for novel transcripts.

**Additional file 8: Table S8.** GO enrichment analysis for *A. cristatum*-specific transcripts.

**Additional file 9: Table S9**. Homologous wheat genes of 6P translocation fragment-specific transcripts in Pubing 3035.

**Additional file 10: Table S10**. Polymorphic molecular marker primer sequences developed between translocation and non-translocation lines.

**Additional file 11: Figure S1**. PCR amplification patterns of polymorphic markers. The red arrows show *A. cristatum*-specific DNA fragments. *M* is a DNA marker; *Lane 1* is *A. cristatum* Z559; *Lane 2* is Pubing 3035; *Lane 3* is Fukuho.

## Data Availability

The sequence data have been deposited in the NCBI under BioProject accession number PRJNA534411.

## Abbreviations

*Bgt*: *Blumeria graminis* f. sp. *tritici*
CCS: Circular consensus sequence
FDR: False discovery rate
FL: Full-length
FLNC: Full-length, non-concatemer
FPKM: Fragments per kilobase of transcript per million mapped reads
Fukuho: Fukuhokumugi
GO: Gene Ontology
IWGSC: International Wheat Genome Sequencing Consortium
lncRNAs: Long non-coding RNAs
mRNAs: Messenger RNAs
PacBio: Pacific Biosciences
RNA-Seq: RNA sequencing
SMRT: Single-molecule, real-time
SVM: Support vector machine
ZMW: Zero-mode waveguide
