# Supplementary material for: Full-length transcriptome sequences of Agropyron cristatum facilitate the prediction of putative genes for thousand-grain weight in a wheat-A. cristatum translocation line
Source: BMC Genomics. 2019 Dec 27;20:1025. doi: 10.1186/s12864-019-6416-4 (PMC6935218; doi:10.1186/s12864-019-6416-4)
Supplement: Supplementary file 11 — Additional file 11: Figure S1. PCR amplification patterns of polymorphic markers. The red arrows show A. cristatum-specific DNA fragments. M is a DNA marker; Lane 1 is A. cristatum Z559; Lane 2 is Pubing 3035; Lane 3 is Fukuho. [file 12864_2019_6416_MOESM11_ESM.pptx]

## Slide 1
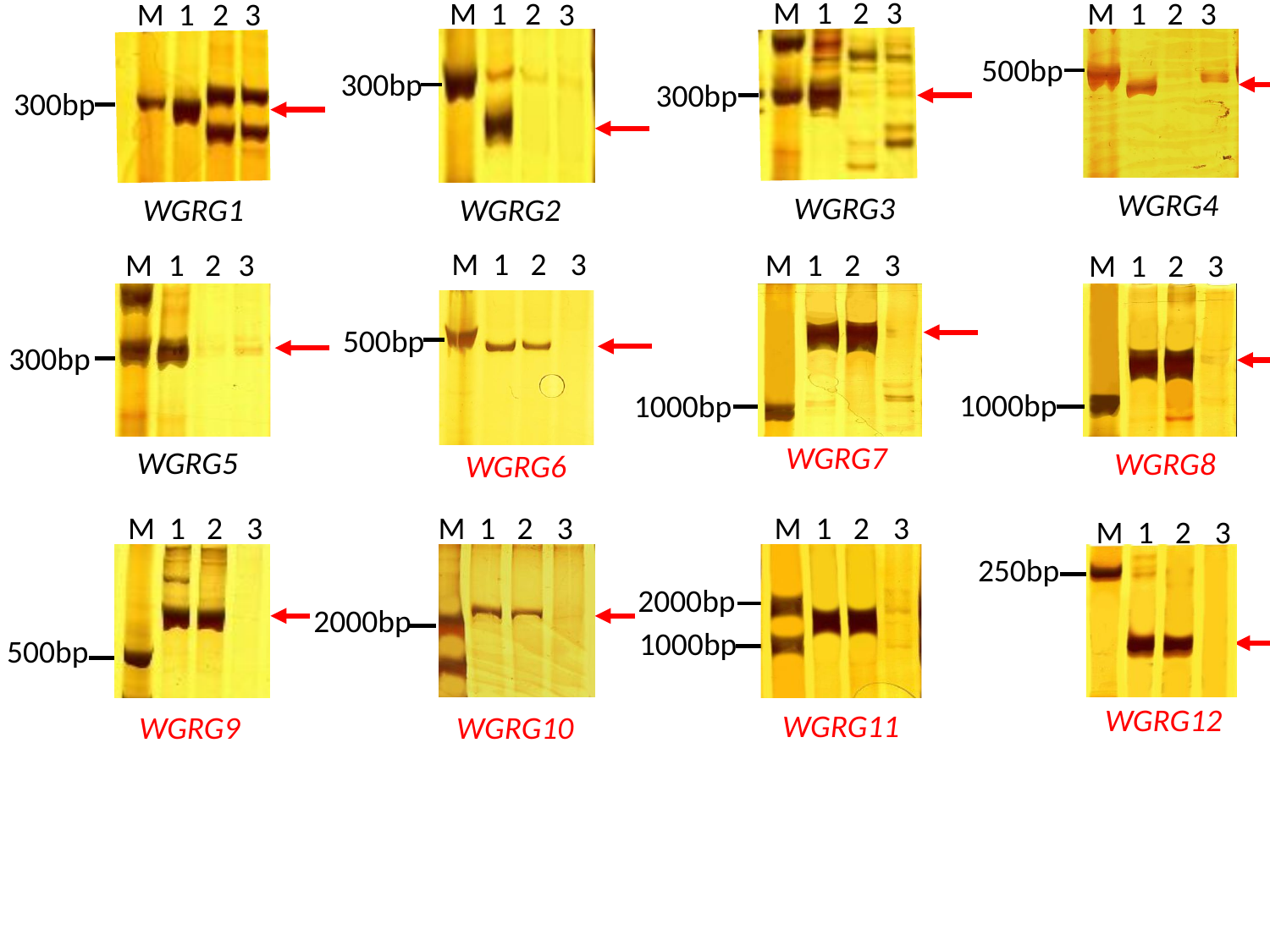

M
1
2
3
WGRG3
300bp
M
1
2
M
1
2
3
500bp
WGRG4
M
1
2
3
300bp
WGRG1
3
300bp
WGRG2
M
1
2
3
M
1
2
3
300bp
WGRG5
M
1
2
3
M
1
2
3
500bp
1000bp
1000bp
WGRG7
WGRG8
WGRG6
M
1
2
3
M
1
2
3
M
1
2
3
M
1
2
3
250bp
2000bp
2000bp
1000bp
500bp
WGRG12
WGRG11
WGRG9
WGRG10

## Slide 2
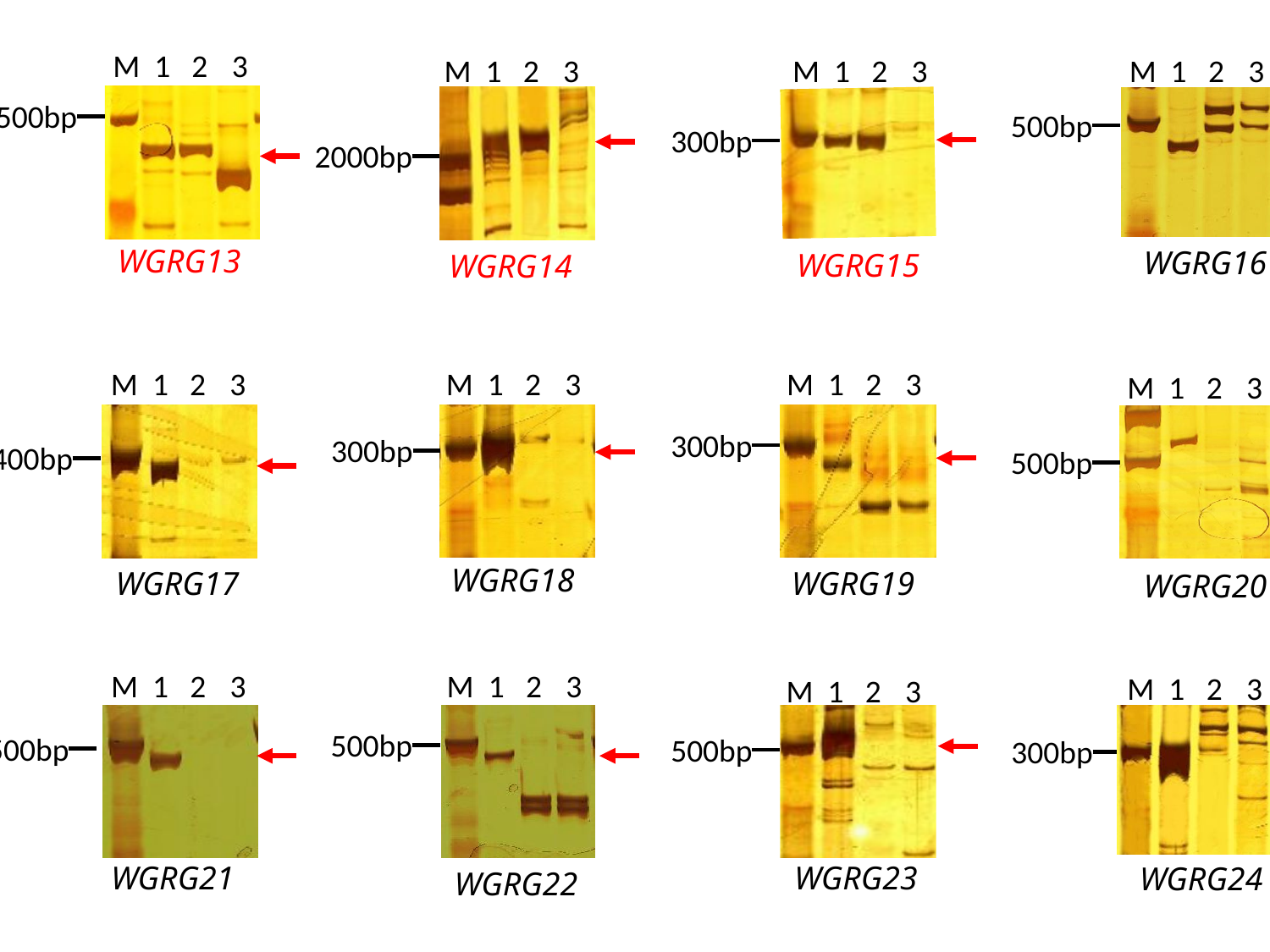

M
1
2
3
500bp
WGRG13
M
1
2
3
M
1
2
3
M
1
2
3
500bp
300bp
2000bp
WGRG16
WGRG15
WGRG14
M
1
2
3
M
1
2
3
M
1
2
3
M
1
2
3
300bp
300bp
400bp
500bp
WGRG18
WGRG17
WGRG19
WGRG20
M
1
2
3
M
1
2
3
M
1
2
3
M
1
2
3
500bp
500bp
500bp
300bp
WGRG21
WGRG23
WGRG24
WGRG22
